# Anthraquinones as Inhibitors of SOS RAS-GEF Activity

**DOI:** 10.3390/biom11081128

**Published:** 2021-07-30

**Authors:** Alberto Fernández-Medarde, Rocío Fuentes-Mateos, Rósula García-Navas, Andrea Olarte-San Juan, José María Sánchez-López, Antonio Fernández-Medarde, Eugenio Santos

**Affiliations:** 1 Centro de Investigación del Cáncer-Instituto de Biología Molecular y Celular del Cáncer (CSIC)-Universidad de Salamanca and CIBERONC, 37007 Salamanca, Spain; rocfuemat@usal.es (R.F.-M.); rosula@usal.es (R.G.-N.); andolasanju@gmail.com (A.O.-S.J.); 2 Biomar Microbial Technologies, Parque Tecnológico de León, Parcela M-10.4, Armunia, 24009 León, Spain; jm.sanchez@biomarmt.com (J.M.S.-L.); a.fernandez@biomarmt.com (A.F.-M.)

**Keywords:** anthraquinones, SOS, RAS-GEF, inhibitors, cancer

## Abstract

Recent breakthroughs have reignited interest in RAS GEFs as direct therapeutic targets. To search for new inhibitors of SOS GEF activity, a repository of known/approved compounds (NIH-NACTS) and a library of new marine compounds (Biomar Microbial Technologies) were screened by means of in vitro RAS-GEF assays using purified, bacterially expressed SOS and RAS constructs. Interestingly, all inhibitors identified in our screenings (two per library) shared related chemical structures belonging to the anthraquinone family of compounds. All our anthraquinone SOS inhibitors were active against the three canonical RAS isoforms when tested in our SOS GEF assays, inhibited RAS activation in mouse embryonic fibroblasts, and were also able to inhibit the growth of different cancer cell lines harboring WT or mutant RAS genes. In contrast to the commercially available anthraquinone inhibitors, our new marine anthraquinone inhibitors did not show in vivo cardiotoxicity, thus providing a lead for future discovery of stronger, clinically useful anthraquinone SOS GEF blockers.

## 1. Introduction

Eukaryotic RAS proteins regulate many cellular processes including proliferation, differentiation, or survival [1] and are continuously cycling between inactive (RAS-GDP) and active (RAS-GTP) conformations. This process is modulated by activating Guanine nucleotide Exchange Factors (GEFs) inducing GDP/GTP exchange, and RAS activation [2], and GTPase activating proteins facilitating GTP hydrolysis [3] and RAS deactivation. SOS GEFs are the most universal and widely expressed RAS-GEF activators in mammalian cells [4,5,6]. Oncogenic RAS mutations cause aberrant regulation of the RAS cycle, leading to human pathologies including hereditary developmental syndromes [7,8] and a wide variety of sporadic cancers [9,10].

The search for RAS inhibitors has been a dominant aim in cancer research for years but no effective drugs able to inhibit these proteins have yet reached clinical use. How-ever, the view that RAS could be “undruggable” [11] has been challenged by recent reports describing the preclinical and clinical characterization of small-molecule inhibitors able to target specific RAS mutations in mutant RAS-driven tumors [11,12,13,14,15,16,17,18,19]. Furthermore, several inhibitors of RAS:SOS interaction have already been isolated [20,21], and BI-1701963 is the first among such inhibitors to reach clinical trials (reviewed in [5]).

Here we describe the identification of several compounds capable of inhibiting the GEF activity of SOS on RAS in an in vitro GEF assay used to screen a collection of about 1000 different compounds including previously known biomedical compounds and new, untested compounds of marine origin. Strikingly, the chemical structures of all positive hits identified in our screening of both libraries shared related chemical structures belonging to the anthraquinone family, which has long been known for its anticancer activity [22].

Our data uncovers a previously unknown mode of action of specific members of the anthraquinone family which may be useful for design of novel therapeutic drugs against mutant RAS-driven cancers.

## 2. Materials and Methods

### 2.1. Compound Libraries

Two separate libraries were screened in this study: (i) the Small-Molecule Resource (SMR) library of the NIH’s National Center for Advancing Translational Sciences (NCATS) (https://ncats.nih.gov/smr, accessed on 23 March 2020), containing 707 compounds used commercially for several purposes and released to find new pharmaceutical uses; and (ii) a collection of 220 compounds of marine origin provided by Biomar Microbial Technologies (Biomar MT, León, Spain) (https://biomarmt.com/, accessed on 24 March 2020). 

### 2.2. Gene Constructs and Protein Purification

A construct containing SOS1 catalytic domain, (amino acids 564-1049, SOS-CAT) cloned in pET15 vector, was kindly supplied by Dr Zheng [23]. Full length Wild-type HRAS, NRAS, KRAS4A and KRAS4B cDNAs inserted into the pHTP1 vector were obtained from NZYtech (Lisbon, Portugal).

For protein purification, 400 mL bacterial cultures were grown to OD600 nm = 0.2 (SOS-CAT), 0.5 (HRAS), 0.8 (NRAS), 0.6 (KRAS4A) or 0.4 (KRAS4B); and stimulated with 1 μg/mL Isopropyl-β-D-1-thiogalactopyranoside: overnight at 30 °C (SOS-CAT), 7 h at 30 °C (HRAS), 9 h at 37 °C (NRAS and KRAS4A) or 4 h at 37 °C (KRAS4B). Cultures were centrifuged 5 min at 8000× *g* and resuspended in lysis buffer (20 mM Tris-ClH pH 8.0, 50 mM NaCl, 5 mM MgCl_2_) plus 10 mM imidazole and sonicated with 2-min pulses, 5 times, on ice. Cellular debris was eliminated by 20-min centrifugation at 20,000× *g* and supernatant was incubated with Ni-NTA Agarose (ThermoFisher scientific, ThermoFisher scientific, Waltham, MA, USA) 2 h at 4 °C. Beads were washed twice in lysis buffer plus 20 mM imidazole and eluted in 400 μL lysis buffer plus 250 mM imidazole. Proteins were aliquoted and stored in liquid nitrogen.

### 2.3. In Vitro GEF Assays

Screenings were performed using a fluorescence-based assay (adapted from [23]) to quantitate the exchange activity of the SOS-CAT on RAS. Briefly, 200 pmoles purified HRAS protein were incubated with 50 pmoles BODIPY^®^-FL-GDP (ThermoFisher scientific) 1 h in the darkness. To a flat-bottom 96-wells black plate we added reaction buffer (20 mM Tris-ClH pH 7.5 + 150 mM NaCl + 1 mM MgCl_2_ + 0.01% NP40 + 1 mM DTT) to a final volume of 100 μL (volume variable depending on protein concentration), 10 μL 1 mM GTP, either 5 μL of 200 μM (NACTS library) or 1 μL of 1 mM (Biomar MT collection) compound solutions (not to positive controls) were added for the screening reactions, adding 50 pmoles SOS1-CAT (not to negative controls) before measurement. The RAS-BODIPY-FL-GDP mixture was added, and the reaction was immediately measured in a fluorescent plate reader (TECAN, Männedorf, Switzerland) for 15 min (Exc.485 nm-Em.535 nm). Negative controls with reaction mix minus SOS and/or RAS proteins were performed for the compounds selected in the screening. In addition, 1 μL of 1 mM BI-2852 (Boehringer Ingelheim, Ingelheim, Germany) was used as a positive control of the inhibition on the exchange reaction. The assays for IC50 determinations maintained similar concentrations of RAS, SOS1, BODIPY^®^-FL-GDP and GTP and final volume (100μL) while adjusting the volume of added compound to obtain final concentrations ranging from 0.1 μM to 100 μM. 

For determination of IC50 values, we quantitated the percentage reduction of the initial rate of the GEF reaction caused by the different compound concentrations tested (from 0.1 μM to 100 μM) during the initial 200 s of reaction (ΔF/t; ΔF = total reduction of fluorescence units; t = 200 s). Normalized values of the percentage (%) inhibition produced by each compound concentration (X) tested were calculated according to the formula [100 – [(ΔF/t)_X_ – (ΔF/t)_C−_/(ΔF/t)_C+_ – (ΔF/t)_C−_] × 100 = % inhibition] where the X, C+ and C− underscripts identify parameter values obtained with each compound concentration and their corresponding positive and negative controls. The IC50 assigned to each compound corresponded to the concentration yielding 50% inhibition of the initial reaction rate in each case. 

### 2.4. Compounds

For the cytotoxicity and in vivo toxicity studies Epirubicin (99.77% purity), Doxorubicin (99.82% purity) and Idarubicin (99.83% purity) were purchased from Selleckchem (Houston, TX, USA). CL0292 (97.3% purity) and CL0294 (94.5% purity) were obtained from Biomar MT (León, Spain). Purity of commercial compounds was determined by HPLC, and NMR as shown in Selleckchem webpage. Purity of Biomar compounds was measured by HPLC. Briefly, 2.5 μL of each compound (0.5 mg/mL) were injected into a Kinetex 2.6 u EVO C-18 100A 4.6 × 50 mm S/N 774392-7 chromatographic column. For CL0292, MeOH:Water (+0.04% TFA) was used as the mobile phase. A gradient of 15–100% of MeOH was applied for the initial 15 min., followed by 5 min. of 100% MeOH and 5 min. of a reverse gradient of 100%-15% MeOH followed by a 5 min. postrun. For CL0294, a mobile phase of MeCN:Water (+0.04% TFA) was used. After sample injection a gradient of 15–100% MeCN was applied for 20 min., followed by 5 min. of a reverse gradient 100–15% MeCN and a postrun for another 5 min. A flow of 0.5 mL/min. was used for both compounds.

### 2.5. Pull-Down Assays

Assays to detect in vivo activation of RAS proteins were performed as previously described [24]. Briefly, total protein extracts from serum-starved WT and SOS2 KO immortalized mouse embryonic fibroblasts (MEFs) were treated with the inhibitors (CL0292 and BI-2852 5 μM; Doxorubicin 0.3 μM; Idarubicin 5.75 μM) for 5 min, and stimulated another 5 min with 10% FBS. These extracts were incubated for 30 min with GST-beads loaded with RAF1 RAS binding domain (RBD) and washed twice with lysis buffer (MLB 1X: 25 Mm HEPES, 150 Mm NaCl, 1% Igepal, 10 mM MgCl_2_, 1 mM EDTA and 2% glycerol). SDS-PAGE loading buffer was added to both pull-down beads and total cell extracts and samples were loaded onto 12% denaturing Polyacrylamide gels, run at 80 V for two hours, transferred, blocked in 5% skim milk with phosphatase inhibitors and incubated with an anti-RAS mouse monoclonal antibody (1:1000 in 5% BSA. Millipore Clone RAS10, 05-516) overnight at 4 °C. Membranes were then washed three times for 5 min in TTBs, incubated with the secondary antibody (1:10,000 in 5% skim milk, ThermoFisher scientific, goat anti-mouse Dy Light 800. SA5-35521) for 1 h at room temperature, washed again three times for 10 min and developed in a LI-COR Odyssey infrared imaging system (LI-COR Biosciences). The intensity of the bands was quantified using the Image-J software and ratios between RAS-GTP and total RAS were calculated and normalized against samples without inhibitors.

### 2.6. Cell Lines

For the pull-down assays we used WT and SOS2 KO MEFs isolated from our WT and SOS2 KO mice [25] and immortalize as previously described [26]. In addition, the compounds were tested for growth inhibition using lung cancer cell lines (NCI-H441 and PC9 [27,28]) and pancreatic cancer cell lines (Hs776, HPAF-II, PANC1 and SKPC [29,30,31,32]). All cell lines but HPAF-II were grown in DMEM with 10% FBS, 2 mM glutamine, 100 UI/mL Penicillin and 100 μg/mL Streptomycin at 5% CO_2_ and 37 °C HPAF-II was grown in RPMI 1640 with the same additives and conditions. Measurement of growth inhibition was monitored using Alamar blue assays (ThermoFisher scientific) according to manufacturer’s instructions. 

### 2.7. Toxicity Assays in Mice

Laboratory mice were handled according to EU and Spanish guidelines for the use of animals in research. Experimentation was approved by the Centro de Investigación del Cáncer Bioethics committee. WT C57Bl6 mice aged 3 months were treated intraperitoneally with the compounds. Cumulative doses: Doxorubicin, 15 mg/Kg; Idarubicin, 8.4 mg/Kg; CL0292 and CL0294, 12 mg/Kg; Vehicle (Doxorubcin and Idarubicin): Saline solution 0.9% NaCl. Vehicle (Biomar MT compounds): 40% PEG, 10% EtOH, 50% dH_2_O. Animals were weighted, monitored with a Small Animal Physiological Monitoring System (Harvard Apparatus) following manufacturer’s instructions, sacrificed after 12-day treatment, and analyzed for histological alterations.

## 3. Results

### 3.1. Library Screenings

**Figure 1:**
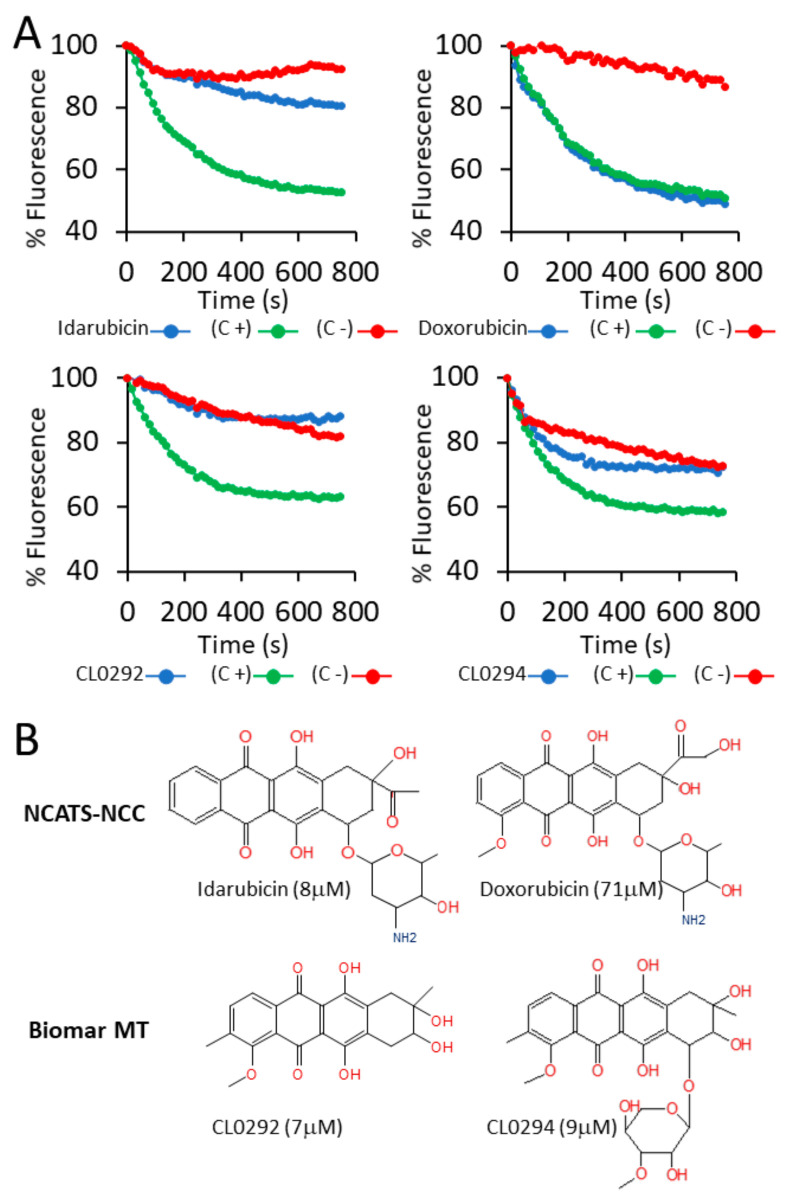
Anthraquinone inhibitors of SOS GEF activity. (**A**) Representative fluorescence tracings of GEF assays performed as described in M&M in the absence (green, positive control, C+) or the presence (blue) of the indicated 4 compounds (10 µM) selected in the screening. As negative controls, the same reactions were carried out in the absence of externally added SOS (red, negative control, C−). Horizontal time scale represented in seconds. Among almost 1000 compounds tested, only Idarubicin, CL0202, CL0294, and to a lesser extent Epirubicin (not shown), sharing a similar anthraquinone structure, showed inhibition in the three separate replicas performed for the screening. Doxorubicin, another molecule with a similar structure but inactive in GEFs assays at the concentration tested, is shown for comparison. (**B**) Chemical structures of the compounds assayed and selected in our screening of the NIH-NCATS library and the Biomar MT library that showed highest inhibitory activity in our in vitro SOS GEF assays. All compounds shared the same anthraquinone ring structure, differing only in the substitutions on the ring. Doxorubicin, which was basically inactive on SOS exchange reaction at the concentration used in the screening (see Figure 1A), has been used as a control throughout this work.

707 compounds from SMR-NCATS and 220 compounds from Biomar MT were analyzed in our fluorescent GEF assay. Using assays in triplicate to confirm positive hits in our screenings, we identified two compounds from the SMR-NCATS library (Idarubicin and Epirubicin) and two compounds from Biomar MT (CL0292 and CL0294) that consistently showed inhibition of SOS GEF activity towards WT HRAS with micromolar IC50s values (Figure 1A; not shown). Although Idarubicin and Epirubicin are known to belong to the same family of anthraquinone compounds [22], the structural analysis and characterization of the Biomar MT CL0294 and CL0292 compounds revealed also similar, related anthraquinone structures (Figure 1B).

Since the ~1000 different compounds in our libraries were not selected based on chemical structure, the anthraquinonic structure of the inhibitors identified in our screenings is not a random coincidence and points to a new functional role for some members of the anthraquinone family as inhibitors of RAS activation by SOS. Notably, this inhibitory action is not a general feature of all anthraquinones since Doxorubicin, a widely used anthraquinone in cancer research [33], as well as several other anthraquinone compounds from the Biomar MT collection, failed to inhibit our SOS RAS-GEF assays at the concentration used in the screening (Figure 1; not shown), suggesting that only certain substitutions on the anthraquinone ring render compounds capable of inhibiting the exchange activity (Figure 1B). These anthraquinones had no effect on the fluorescence emitted by BODIPY^®^-FL-GDP when the reaction components were added to the plates and measured in the absence of proteins (data not shown), which indicates a specific effect on the exchange reaction.

Although our initial screening was performed using isolated HRAS protein, similar degrees of inhibition were observed with the identified compounds when purified NRAS, KRAS4A or KRAS4B were used in the assay (Table 1), documenting the ability of these anthraquinone compounds to block the activation of all canonical RAS isoforms by SOS.

### 3.2. Ras Activation Is Inhibited by the Anthraquinones In Vivo

**Figure 2:**
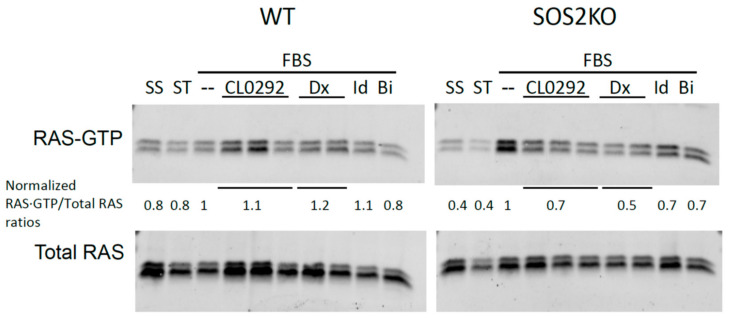
Pull-down assays using the inhibitors of the exchanger reaction on MEFs. Inhibition of Ras activation was observed only in the absence of the SOS2 protein, indicating that the anthraquinones used in this study selectively inhibit RAS activation by SOS1. Quantifications of the band intensities were converted into RAS·GTP/Total RAS ratios and normalized against the value obtained in stimulated cell without the inhibitors. Ratios between levels of RAS-GTP and total RAS are indicated for all assays. SS: Steady State; ST: Starved; --: DMSO; Dx: Doxorubicin; Id: Idarubicin; Bi: BI2852. (Quantification and statistics (SEM): (WT) SS *n* = 2, 0.8 ± 0.05; ST *n* = 2, 0.8 ± 0.18; CL0292 *n* = 6, 1.1 ± 0.13; Doxorubicin *n* = 4, 1.2 ± 0.22; Idarrubicin *n* = 2, 1.1 ± 0.24; BI-2852 *n* = 2, 0.8 ± 0.01. (SOS2 KO) SS *n* = 2, 0.4 ± 0.18; ST *n* = 2, 0.4 ± 0.26; CL0292 *n* = 6, 0.7 ± 0.10; Doxorubicin *n* = 4, 0.5 ± 0.10; Idarrubicin *n* = 2, 0.7 ± 0.13; BI-2852 *n* = 2, 0.7 ± 0.24).

To see if the inhibition observed in vitro was translated to a similar blockage of RAS activation in vivo, we used the anthraquinones described above to treat mouse embryonic fibroblasts (MEFs). In addition, we used BI2852 a known inhibitor of SOS1:RAS interaction as a positive control [34]. Interestingly, none of the compounds showed inhibition of overall RAS activation levels in WT MEFs (see Figure 2, left). As our compound screenings were performed using the isolated catalytic domain of SOS1, and WT MEFs express both SOS1 and SOS2 isoforms, we decided to analyze if the lack of inhibition was due to a potential compensatory activation mediated by SOS2 in the absence of SOS1. For this purpose, we tested the inhibitors on MEFs lacking SOS2 [25]. As shown in Figure 2, when SOS2 was removed from the fibroblasts the anthraquinone inhibitors were able to reduce overall RAS activation by FBS, demonstrating that the compounds selected in the screening are specific inhibitors of SOS1.

### 3.3. Inhibition of Growth of Cancer Cell Lines 

**Figure 3:**
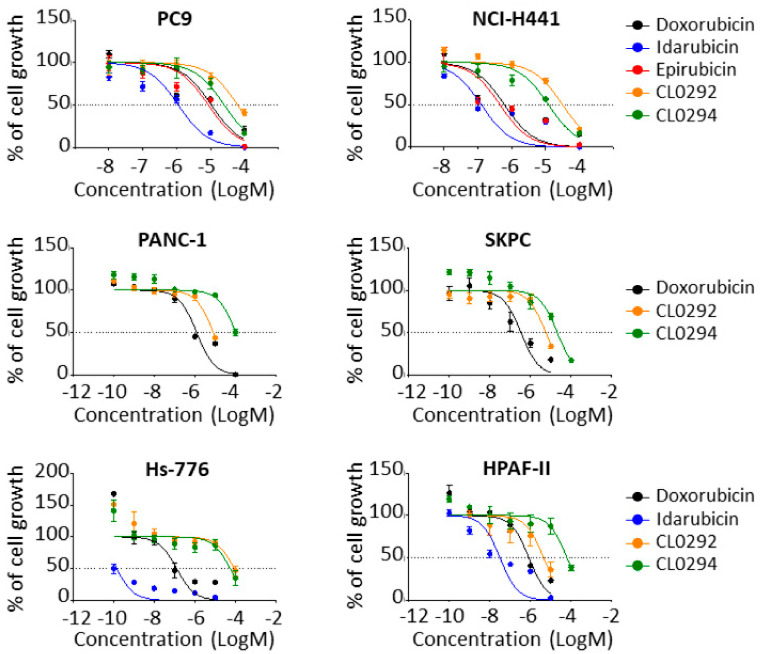
Inhibition of growth of cultured cancer cell lines. The indicated cell lines were exposed to increasing concentrations of Doxorubicin (used as positive control of inhibition), Idarubicin, Epirubicin, CL0292 and CL0294 for 48 h and cell survival was assessed using AlamarBlue^®^ assays as described in Materials and Methods. Results are expressed as percentage of cell growth compared to untreated cells.

We also tested the compounds uncovered in our screening for their ability to inhibit growth of various cancer cell lines using the known anticancer anthraquinone Doxorubicin as a control (Figure 3). In lung cancer cells, the Doxorubicin, Idarubicin, and Epirubicin showed significantly higher inhibitory activity than the CL0292 and CL0294 marine anthraquinones. Interestingly, the growth inhibitory activity of all our anthraquinone compounds appeared to be stronger against the NCI-H441 cell line (harboring a KRAS G12V mutation [27]) than against PC9 cells, harboring WT KRAS [28] (Figure 3, Table 2).

Doxorubicin and Idarrubicin showed also significantly higher activity than the CL0292 and CL0294 compounds when assayed on pancreatic tumor cell lines harboring different KRAS mutations (KRAS G12D in SKPC, PANC1 and HPAF-II [29,30,31], and KRAS Q61H in Hs-776 [32]). In contrast to their behavior in lung cell lines, CL0292 showed higher inhibitory activity than CL0294 when assayed on the pancreatic lines (Figure 3, Table 2). These data document the ability of our newly identified marine anthraquinone SOS GEF inhibitors to prevent the growth of tumoral cells carrying either WT or mutant KRAS oncogenes.

### 3.4. Toxicity Studies in Mice

**Figure 4:**
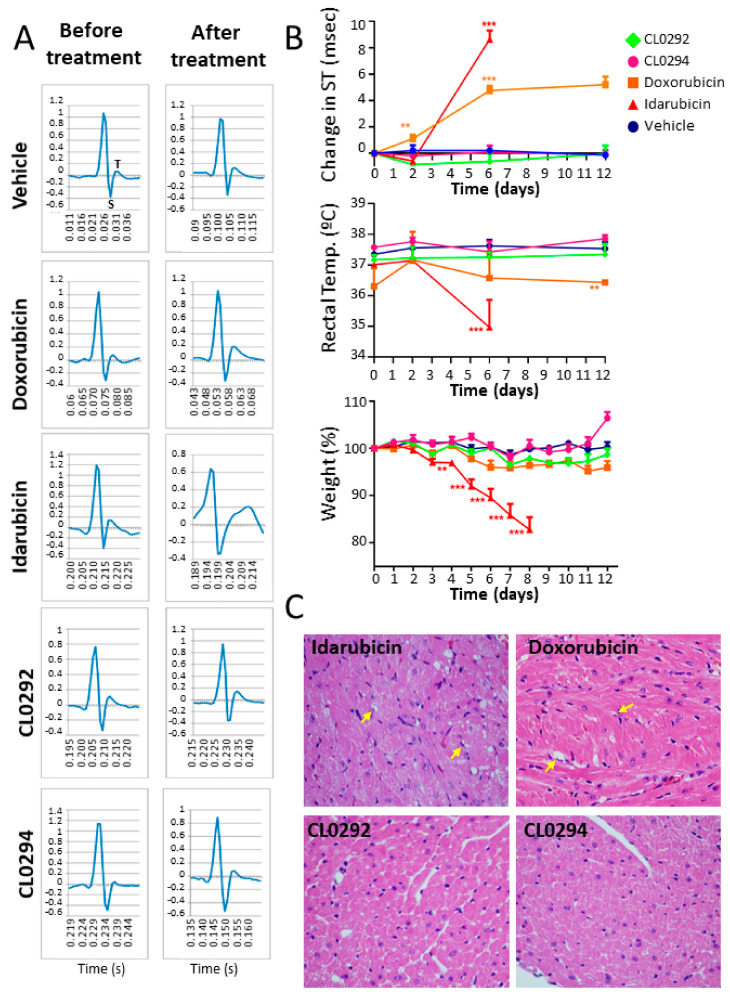
Toxicity assessment in mice. (**A**) Electrocardiograms and ST changes from mice untreated and treated for 6 (Idarubicin) or 12 days (all other compounds) with the indicated vehicle or anthraquinone compounds from our screening studies. The ST interval reflects the repolarization of the cells and is measured from the deepest valley (S) in the ECG to the top (T) of the round crest that follows. Alterations in the ST interval reflect defects in cardiomyocyte function. (**B**) Evolution of the ST interval values, body temperature and weight changes for the 12 days treatment (*n* ≥ 3). As shown, Doxorubicin and especially Idarubicin-treated mice showed marked decreases in body weight and body temperature and these changes were not observed in CL0292 or CL0294 treated rodents. ** *p* < 0.01, *** *p* < 0.001 against all other phenotypes (ANOVA Bonferroni). (**C**) Hematoxylin/Eosin staining of heart tissues from animals treated with the anthraquinone inhibitors isolated in our screening. Doxorubicin treatment was used here as a positive control. Arrows point to cell vacuolization occurring in the left ventricular area.

Anthraquinones have been used for years as anticancer agents but severe secondary effects due to their toxicity have been described [35]. In particular, Doxorubicin and Idarubicin are known to induce high rates of cardiomyopathy [36,37]. To analyze the toxicity of the compounds identified in our screening, we treated wild-type C57Bl6 mice with the compounds previously tested in cell lines (Figure 3), using concentrations previously shown to induce cardiotoxicity in mice [38,39]. As expected, Idarubicin and Doxorubicin produced marked cardiac toxicity, with significantly increased ST-intervals in electrocardiograms of the treated mice, as well as markedly decreased weight and lower body temperature than the controls (Figure 4A,B). Indeed, the Idarubicin-treated mice had to be sacrificed in advance, after six days of treatment, due to its high toxicity (Figure 4A). The Idarubicin-treated and Doxorubicin-treated mice displayed also clear heart histological alterations, showing marked myocyte cytoplasm vacuolization in the left ventricle (Figure 4C) and some Idarubicin-treated mice also showed emphysema (data not shown). In sharp contrast, no signs of toxicity were found in the mice treated with CL0292 or CL0294, which showed normal cardiac and respiratory values (Figure 4A), no loss of weight, decreased body temperature or emphysema (Figure 4B). Furthermore, histological analysis of the heart of mice treated with CL0292 or CL0294 did not find any gross anomalies or cytoplasm vacuolization of myocytes (Figure 4C). Our data document that our CL0292 and CL0294 marine anthraquinone compounds, inhibit SOS GEF activity in vitro, without displaying any detectable toxicity on mice. 

## 4. Discussion

To our knowledge, this is the first report describing anthraquinones as capable of inhibiting the in vitro RAS-GEF activity of the cellular SOS1 exchanger. Recent reports have described the identification of novel inhibitors of RAS activation by cellular GEFs (reviewed in [18,40]) but none of them belong to the anthraquinone family of compounds. Consistently, a wide spectrum of anthraquinone compounds with different biological activities have been previously used as anticancer agents [22,25,41], but none of them were described as possessing this type of specific SOS GEF inhibitory activity. Thus, our data adds a new mechanism of action to the list of anticancer actions played by different members of this family of drugs. Since Doxorubicin and other Biomar MT anthraquinones did not inhibit SOS GEF activity in our assays at the concentration used in the screening, it is apparent that only certain modifications of the anthraquinone ring are able to confer the ability to inhibit SOS GEF activity. Our data show that these compounds can inhibit the GEF exchange reaction in vitro and in vivo. Somewhat surprisingly, all anthraquinone inhibitors failed to show inhibition of overall RAS activation in vivo when WT MEFs were used in the assay but showed inhibitory activity when analyzed on SOS2-KO MEFs. This can be explained by a specific effect of these inhibitors on SOS1. In WT MEFs (expressing both SOS1 and SOS2), SOS2 would compensate for SOS1 inhibition resulting in normal levels of RAS activation. In contrast, ablation of SOS2 would eliminate such compensatory effect, thus allowing the detection of the inhibition of RAS activation mediated by the anthraquinone SOS1 inhibitors. In addition, although RAS activation is blocked by all three anthraquinones tested, the highest inhibition was achieved with the commercial Doxorubicin and Idarubicin. This can be explained by the process of optimization followed in the development of these drugs, and the fact that the non-commercial CL0292 is less stable (data not shown) and has not been optimized for better cellular uptake, which could account for the differences observed between the data obtained in vitro and in vivo. In this regard, we cannot formally discard that some of the inhibitory effects that these compounds have on cell growth could be due to their ability to inhibit other targets in vivo [42]. This could account for the differences in potency observed between the in vitro assays, where CL0292 and CL0294 have a similar efficiency than Idarubicin in blocking the exchanger reaction, and the data obtained with the cell lines, in which commercial anthracyclines show stronger inhibition. In addition, the inhibitory activity on the exchange reaction could explain the better efficacy of Idarubicin compared to Doxorubicin in inhibiting cell growth, adding inhibition of RAS activation to its effect on topoisomerase II.

Despite the absence of secondary effects observed in mice treated with the CL0292 and CL0294 compounds, their IC50 values for in vitro inhibition of SOS GEF activity or inhibition of growth of cultured cancer cells are still too high. In addition, their lack of toxicity in vivo could be attributable to reduced efficacy inhibiting cell growth, low absorption, or fast rates of elimination. Thus, future work is needed to study those possibilities, as well as the efficiency of these compounds regarding tumor growth inhibition. Additionally, to identify inhibitors with better IC50 values, it will be interesting to ascertain the specific structural alterations of the anthraquinone ring that favor the in vitro inhibitory action on SOS GEF activity.

The discovery of the inhibitory activity of anthracyclines on RAS activation opens new avenues in the search for small molecules able to inhibit downstream oncogenic signaling in RAS-dependent tumors and Rasopathies. Our preliminary characterization of the compounds isolated in this screening, supports further efforts aimed at searching for related compounds that maintain absence of toxicity while showing lower IC50 values for inhibiting SOS1-mediated RAS activation or blocking growth of cultured cells from RAS-dependent tumors. It will also be of interest to determine in the future whether our currently identified inhibitors can also target the GEF activity of SOS2 [5], as well as performing similar screenings aimed at identifying further RAS-GEF inhibitors able to discriminate between SOS1 and SOS2. 

## Figures and Tables

**Table 1:** IC50 for RAS isoforms.

Isoform	Compound	IC50 (μM)
HRAS	Idarubicin	8.37 ± 0.94
	Doxorubicin (C−)	70.56 ± 46.15
	CL0292	7.37 ± 1.69
	CL0294	8.89 ± 1.82
NRAS	Idarubicin	7.91 ± 0.66
	Doxorubicin (C−)	44.38 ± 18.07
	CL0292	9.2 ± 0.94
	CL0294	13.94 ± 3.42
KRAS4A	Idarubicin	5.75 ± 1.16
	Doxorubicin (C−)	45.63 ± 16
	CL0292	5.41 ± 1.54
	CL0294	9.14 ± 2.44
KRAS4B	Idarubicin	6.78 ± 2.15
	Doxorubicin (C−)	41.01 ± 23.81
	CL0292	4.98 ± 1.41
	CL0294	8.96 ± 1.48

IC50 values (±SEM) of the anthraquinones analyzed in this work against all four RAS isoforms.

**Table 2:** IC50 values for anthraquinone compounds inhibiting the growth of lung and pancreatic cancer cell lines.

Cell Line	Compound	IC50 (μM)
PC9 Lung	Idarubicin	1.1 ± 0.28
	Epirubicin	8.9 ± 1.37
	Doxorubicin	10.1 ± 1.16
	CL0292	49.5 ± 16.28
	CL0294	25.9 ± 3.52
NCI-H441 Lung	Idarubicin	0.26 ± 0.06
	Epirubicin	6.9 ± 0.8
	Doxorubicin	0.61 ± 0.17
	CL0292	31.8 ± 2.38
	CL0294	12.1 ± 0.58
PANC1 Pancreas	Doxorubicin	1.2 ± 0.16
	CL0292	8.1 ± 0.80
	CL0294	146.1 ± 5.27
SKPC Pancreas	Doxorubicin	0.3 ± 0.02
	CL0292	5.6 ± 0.55
	CL0294	20.7 ± 1.85
HPAF-II Pancreas	Idarubicin	9 × 10^−4^ ± 2.8 × 10^−6^
	Doxorubicin	0.8 ± 0.07
	CL0292	4.4 ± 0.25
	CL0294	62.1 ± 7.99
Hs-776 Pancreas	Idarubicin	0.038 ± 0.001
	Doxorubicin	0.1 ± 0.09
	CL0292	87.2 ± 15.88
	CL0294	55.0 ± 10.54

IC50 values (±SEM) for the anthraquinones analyzed in this work in the inhibition of two lung cancer and four pancreatic cancer cell lines.

## Data Availability

Not applicable.
